# Preparation and Characterization of Injectable Brushite Filled-Poly (Methyl Methacrylate) Bone Cement

**DOI:** 10.3390/ma7096779

**Published:** 2014-09-19

**Authors:** Lucas C. Rodriguez, Jonathan Chari, Shant Aghyarian, Izabelle M. Gindri, Victor Kosmopoulos, Danieli C. Rodrigues

**Affiliations:** 1 Department of Bioengineering, the University of Texas at Dallas, 800 West Campbell Road, Richardson, TX 75080, USA; E-Mails: lxr120230@utdallas.edu (L.C.R.); jxc122130@utdallas.edu (J.C.); sxa117530@utdallas.edu (S.A.); idg130030@utdallas.edu (I.M.G.); 2 Department of Orthopaedic Surgery, University of North Texas Health Science Center (UNTHSC), Fort Worth, TX 76107, USA; E-Mail: Victor.Kosmopoulos@unthsc.edu; 3 Department of Materials Science and Engineering, University of North Texas, Denton, TX 76203, USA

**Keywords:** Poly (methyl methacrylate) bone cement, polymer composites, calcium phosphate, complex viscosity, rheology, compressive strength

## Abstract

Powder-liquid poly (methyl methacrylate) (PMMA) bone cements are widely utilized for augmentation of bone fractures and fixation of orthopedic implants. These cements typically have an abundance of beneficial qualities, however their lack of bioactivity allows for continued development. To enhance osseointegration and bioactivity, calcium phosphate cements prepared with hydroxyapatite, brushite or tricalcium phosphates have been introduced with rather unsuccessful results due to increased cement viscosity, poor handling and reduced mechanical performance. This has limited the use of such cements in applications requiring delivery through small cannulas and in load bearing. The goal of this study is to design an alternative cement system that can better accommodate calcium-phosphate additives while preserving cement rheological properties and performance. In the present work, a number of brushite-filled two-solution bone cements were prepared and characterized by studying their complex viscosity-versus-test frequency, extrusion stress, clumping tendency during injection through a syringe, extent of fill of a machined void in cortical bone analog specimens, and compressive strength. The addition of brushite into the two-solution cement formulations investigated did not affect the pseudoplastic behavior and handling properties of the materials as demonstrated by rheological experiments. Extrusion stress was observed to vary with brushite concentration with values lower or in the range of control PMMA-based cements. The materials were observed to completely fill pre-formed voids in bone analog specimens. Cement compressive strength was observed to decrease with increasing concentration of fillers; however, the materials exhibited high enough strength for consideration in load bearing applications. The results indicated that partially substituting the PMMA phase of the two-solution cement with brushite at a 40% by mass concentration provided the best combination of the properties investigated. This alternative material may find applications in systems requiring highly injectable and viscous cements such as in the treatment of spinal fractures and bone defects.

## 1. Introduction

Acrylic poly (methyl methacrylate) bone cements (PMMA) have been used in a variety of healthcare applications, especially in orthopedics, trauma, and craniofacial surgery. PMMA bone cements provide a multitude of advantageous characteristics such as high strength, bioinertness, biocompatibility and long-term clinical history [1,2]. However, drawbacks related to acrylic cements are typically associated to high curing temperatures, high residual monomer, shrinkage, and poor interdigitation with bone. These have been postulated to lead to bone necrosis and mechanical loosening *in vivo* [3].

Calcium-phosphate (CaP)-bone cements offer several clinical advantages in comparison to traditional all-acrylic poly (methyl methacrylate) (PMMA) formulations. CaP cements promote osteogenesis, osteoconduction, and resorption, which can have fast or slow degradation rates depending on the type of filler used [4,5,6]. Other important characteristics of these cements are low toxicity, adequate setting time, and low exothermal temperatures [5]. On the other hand, current CaP cements on the market (Norian, Bone Source, Mimix, *etc.*) are largely limited to non-load bearing applications due to the lack of mechanical strength [7]. Additionally, the incorporation of CaP additives has typically resulted in increased cement viscosity, difficult handling, and delivery [8,9,10,11,12].

These current challenges encountered with CaP and all-acrylic PMMA-based cement systems demonstrate the need for the development of an alternative bioactive CaP-PMMA composite cement system that exhibits adequate viscosity and ease of injection, while sustaining strength.

Until recently, CaP materials have not been thoroughly investigated as a partial PMMA bone cement substitute or additive [13]. A recent study demonstrated the feasibility of loading CaP fillers into a two-solution PMMA bone cement system. Two-solution bone cements have been previously demonstrated to constitute a versatile alternative to the traditional powder-liquid cement formulations [14,15]. The material has a combination of advantageous properties including high strength, high pseudoplasticity, lower exothermal temperatures, pre-mixing and simple manipulation of the chemical composition. A highly viscous, pseudoplastic cement is of great interest because: (1) the cement will undergo shear-thinning, which will improve interdigitation with cancellous bone while stabilizing flow [6,16,17,18,19]; and (2) highly pseudoplastic cement will simultaneously facilitate flow through needles while inhibiting deleterious extravasation by viscosity recovery at the delivery sites [20,21]. However, limitations of the original all-acrylic two-solution cement included low polymer-to-monomer ratio (P:M), short setting-time, and high residual monomer content. The viscosity of the original cement was manipulated in previous studies by the incorporation of cross-linked PMMA microspheres (μ-TSBC), nanospheres (η-TSBC), and polymer brushes [22,23,24]. Although, viscosity and other properties of the cement were greatly improved with the addition of a cross-linked phase, the synthesis of cross-linked particles and brushes proved to be a complex procedure with low yields. Therefore, the concept of adding a cross-linked phase to optimize cement properties was proved to be difficult and non-advantageous. Besides, it limited any possibilities of adding an additional filler phase, such as calcium phosphate, that could provide bioactivity.

In this study, new composite two-solution bone cements are discussed. The material combines two systems (CaP and PMMA) utilizing the advantages of a pre-mixed cement formulation. To overcome the lack of bioactivity, a CaP phase, more specifically Brushite (CaHPO_4_·2H_2_O), has been incorporated into the cement matrix at varying concentrations (0%–50% by mass). The rheological characteristics and cement flow were characterized in this initial study to investigate the feasibility of developing highly viscous and pseudoplastic composite two-solution cements that could be injected without the aid of high pressure devices. Mechanical strength of these cements was also evaluated. The CaP-containing cement system has been designed to combine the strength of acrylic PMMA with the osteoconductive benefits of CaP compounds [7]. The rheological characteristics of the two-solution system are advantageous because highly pseudoplastic materials can inhibit cement extravasation and its deleterious effects *in vivo*. Ideally, bone cements should be tuned so that under high shear stresses it exhibits low viscosity, to facilitate injection and to adhere well to desired contact surfaces, while still being viscous enough to maintain shape once shear is removed. The ultimate goal is to develop a unique, bioactive, highly-viscous, and easily injectable PMMA-brushite two-solution bone cement for multiple surgical applications.

## 2. Experimental Section

### 2.1. Materials

All chemicals were used as received from manufacturers without any further modification. PMMA (Monomer-Polymer and Dajac Lab, Trevose, PA, USA) (80,000 g/mol) was used as an aid to increase the viscosity (molecular weight) of the cement mixture. Methyl methacrylate (Fisher Scientific, Waltham, MA, USA) was used as the monomer for the mixture. Benzoyl Peroxide (BPO) (Sigma Aldrich, St. Louis, MO, USA) and *N*,*N*-dimethyl p-toluidine (DMPT) (Sigma Aldrich) were used as the initiator and activator of the free radical polymerization reaction. Brushite (Fisher Scientific) was used as the calcium phosphate filler additive/substitute in the cements. The calcium phosphate dibasic dihydrate (Brushite) (Sigma Aldrich CAS#7789-77-7) utilized for the current study has an unreported particle size. The unpredictable water uptake of the material results in the formation of large clumps. The manufacturer (Sigma Aldrich) does not quantify the specific particle size for this material due to the variability in water content, which can yield variations in particle size.

### 2.2. Cement Preparation

The preparation of the standard TSBC followed protocols described in previous studies [14,20]. In summary, the two-solution cement is a pre-mixed system where all the components that make up the mixture are mixed, swollen, and stored in double-barrel cartridges. One side of the cartridge contains a mixture of the monomer (MMA), the polymer (PMMA), filler (Brushite), and initiatior (BPO), while the other side of the cartridge contains the same elements but instead of BPO the accelerator is added in the mixture. This cement is ready for use and can be mixed and dispensed on demand by using disposable static mixing elements. In preparing each cement batch, a fixed concentration of initiator (BPO) and accelerator (DMPT) was used (1.25% w/v and 0.7% v/v, respectively) (w/v referring to the percent amount of the compound in relation to the PMMA weight or MMA volume). Standard all-acrylic cement cartridges (referred to as “control”) were prepared by mixing the monomer (MMA) and the polymer (PMMA) in a 0.9:1 powder-to-liquid (P:L) ratio (9 grams of PMMA and 10 mL of MMA) without any filler added. The Brushite-filled cement cartridges were prepared by adding the fillers in concentrations of ~10%–50% by mass. The cement compositions investigated are described in Table 1. Fillers were incorporated into the system in two different methods. Initially, the CaP fillers were added to the system while leaving all other components the same (referred to as composite bone additives—“CBA”). Next, CaP fillers were added in varying concentrations while removing dry polymer (PMMA) from the system (referred to as composite bone substitutes—“CBS 25” and “CBS 50”) to offset the overall powder content in the mixture. CBS 25 refers to a cement system with 25% of the dry polymer phase removed, and CBS 50 refers to a system with 50% of the dry polymer phase removed. The nomenclature for the cement compositions is summarized in Table 1.

**Table 1:** Description of cement formulations prepared and % mass Brushite filled into each composition investigated.

Composition	Powder: Liquid	Definition of Types of Compositions Investigated	
		Description	Percent Mass Brushite
Control	0.9:1	No polymer removed and no filler added	0%
CBA	1:1–1.5:1	Filler added to original composition	10%–18%
CBS 25	1:1–2:1	25% polymer phase removed prior to filling	10%–40%
CBS 50	1:1–2:1	50% polymer phase removed prior to filling	20%–50%

The additives/substitutes were well dispersed into the polymer phase of the cement prior to mixing with monomer, activator, and initiator. Each mixture (activator side *versus* initiator side) was mixed separately to prevent early initiation. The cement mixtures were then loaded into double barrel cartridges and mixed thoroughly for 18 h at 125 rotations per minute (RPM). The compositions were then stored at 4 °C for 72 h prior to testing to allow for the components of the cement mixture to reach full swelling. The final powder-to-liquid ratio (P:L) of the cement compositions investigated ranged from 1:1 to 2:1.

### 2.3. Handling and Cement Flow

Clumping was investigated by monitoring the extrusion force during material injection at a constant rate with a Mechanical Testing System (MTS Bionix, Model 370, Eden Prairie, MN, USA). Cement cartridges were loaded in the MTS system with their plungers and mixing nozzles oriented vertically in relation to the load point. Cement cartridges were subject to an axial displacement of 20 mm/s. This rate was selected based on human factors to simulate the speed at which a user would extrude the material from the cartridge. Because clumping could possibly be affected by the rate of extrusion and the pressure built in the cartridge, these parameters were standardized for the experiment. The resulting force was monitored, and clumping was defined as an increase in extrusion force during the injection. A homogenous mixture with no clumping or filter pressing would have a uniform force during and throughout the injection. Cement flow was examined by creating a 2 mm diameter × 5 mm depth cylindrical defect in a solid polyurethane foam model (Sawbones^®^, density 480.55 kg/m^3^, Vashon Island, WA, USA) and injecting the investigative material into the defect. Post-cure, the sealed defect was sectioned along the midline and imaged using 3D Digital Microscopy to determine extent of sealing (Keyence, VHX 2000, Osaka, Japan).

### 2.4. Rheology

Rheology tests were performed using a Discovery HR-3 Hybrid Rheometer (TA Instruments, New Castle, DE, USA). All cement compositions were removed from 4 °C and allowed to reach room temperature for 2 h prior to testing as per ASTM Standard F451-08 [25]. Static mixing nozzles (Ellsworth Adhesives, Germantown, WI, USA) were used to mix the two components of the material and elute the cement mixture onto the bottom rheometer plate. Given the size of the filler materials used in the cement mixture, disposable parallel plate geometries were selected for this study. Parallel plates with a diameter of 25 mm were used with all cement compositions evaluated. Material volumes of 0.5 mL and a geometry gap of 1000 ± 300 µm (at least one order of magnitude larger than the largest particle present in the investigative material) were set as testing standards [26]. Materials were also tested at a 1 mL volume and 2000 µm geometry gap to ensure the data was not dependent on dimensional parameters. After examination of the results obtained with different geometry gaps and volumes, it was determined that the measurements were independent of this variable and the 1000 µm geometry gap with 0.5 mL sample volume were adopted for the remaining samples throughout the test.

**Figure 1:**
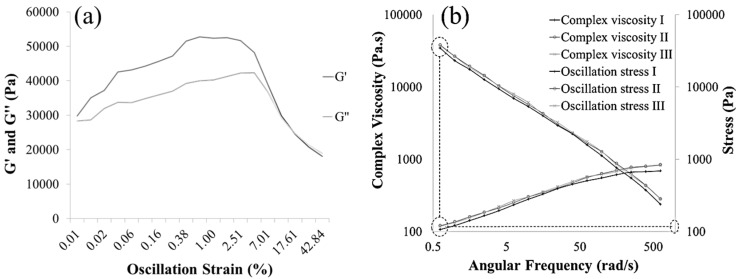
The graphical representations of (**a**) the acquisition of the linear viscoelastic region and (**b**) the extrusion stress of the materials examined. The near-zero complex viscosity was correlated to the stress value at that point to yield extrusion stress as indicated in the graph (**b**) with dashed lines.

Oscillation frequency was selected over continuous shear due to the viscoelastic behavior of the material and observation of the Weissenberg effect in steady shear stress mode. Dynamic rheometry was thus adopted as the means of characterizing the material properties. The testing parameters remained constant for each composition tested. Testing temperature was set to 24 °C with no soak time. Continuous oscillation frequency was applied using a logarithmic sweep function with a frequency range of 100–0.1 Hz. Percent strain was constrained to 0.5% for each test after an amplitude sweep was performed to detect the range of the linear viscoelastic region (LVR) as illustrated in Figure 1a,b. Parameters investigated included storage and loss moduli (G′ and G″), and complex viscosity (η*) as functions of time and frequency. All formulations investigated were independently tested in triplicate and then averaged.

### 2.5. Extrusion Stress (σ_extrusion_)

Yield stress is an important parameter because it gives an estimate of the pressure or stress required for injection or extrusion of a material. However, pseudoplastic materials demonstrate no true yield stress, meaning they are capable of flowing instantaneously following stress application. Therefore, they are defined as behaving in a non-Newtonian manner due to display of shear-thinning effects. Thus, the extrusion stress (σ_extrusion_) of each of the formulations developed was investigated. The extrusion stress was defined as the oscillatory stress value recorded at the maximum viscosity (lowest angular frequency, which elicited an oscillatory stress, at 0.63 rad/s, Figure 1b) of the material during frequency sweep experiments. Because this oscillatory stress refers to the stress at the maximum viscosity of the cement composition, it is directly related to the maximum stress required to begin extrusion of the cement mixture from its cartridge. All formulations were tested in triplicates with each sample being obtained from a different batch.

### 2.6. Compressive Strength

A MTS Bionix mechanical testing system (Model 370, MTS Systems Corporation, Eden Prairie, MN, USA) was used to perform the mechanical testing experiments to investigate the compressive strengths of the various bone cements prepared. The effect of CaP fillers was explored by adding Brushite to the compositions (in concentrations of 0%–50% by mass). Teflon molds were used to mold symmetrical cylinders for testing. The pellets had dimensions of 6 mm of diameter by 12 mm length. The time lapse between sample molding and mechanical testing was seven days while the samples were stored at room temperature.

Samples were loaded onto the center ring of compression platens and then subjected to a pre-load of 0.01 kN before beginning the axial ramp at 20.0 mm/min to a final specimen height of 2 mm [25]. Axial force and axial displacement were recorded and converted to stress and strain using the dimensions of the individual samples. Each composition was tested with five samples and the average of these samples was recorded with standard deviations. The compressive strength of the material was defined as the stress at 2% offset from the stress *versus* strain curve, or the stress at the upper yield point (whichever occurred first).

### 2.7. Statistical Analysis

A one-way Analysis of Variance (ANOVA), as well as multiple comparisons across values test was performed to show differences among the groups compared at a confidence interval of 95%. MATLAB (R2012a, Natick, MA, USA) was used to perform the statistical analysis. All compositions were evaluated against the control.

## 3. Results

### 3.1. Handling and Cement Flow

**Figure 2:**
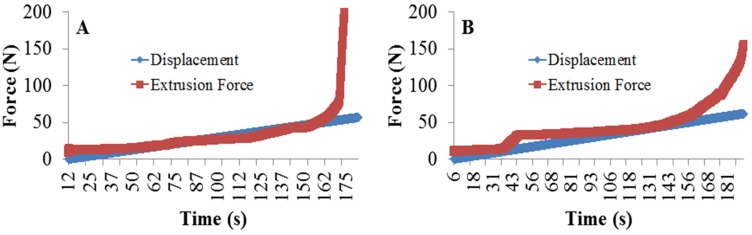
The extrusion force monitored over time for (**A**) composite bone substitute (CBS) 50 40% filled compositions demonstrating a lack of clumping evidenced by uniform stress required to extrude the material from its cartridge; and (**B**) CBS 50 50% filled compositions demonstrating clumping evidenced by the increased extrusion force in the first third of the experiment.

Both of the CBA compositions, as well as the highly-filled 2:1 powder-to-liquid ratio compositions (CBS 25 and CBS 50), exhibited clumping near the last 1/4–1/3 of the cartridge (Table 2). This was observed by the dramatic increase in force generated near the end of the cartridge. These formulations were unable to be fully extruded from their cartridge indicating clumping or cement de-mixing. Other formulations, as indicated in Table 2, exhibited adequate handling and extrusion and were therefore further characterized. Figure 2 illustrates the uniform force distribution throughout the cartridge during material extrusion of a CBS 50 40% filled cement.

**Table 2:** Pseudoplasticity, extrusion stress, and clump formation of compositions investigated.

Composition	Complex Viscosity Slope	Mean Extrusion Stress (Pa) ± Standard Deviation	Clumping Observed
1:1 (P:L)			
Control (0.9:1)	−0.7	115.5 ±7.3	No
CBA 10% CaP	−0.7	102.3 ± 11.2	Yes
CBS 25 12% CaP	−0.7	27.1 ± 4.5	No
CBS 50 24% CaP	−0.8	26.2 ± 3.3	No
1.5:1 (P:L)			
CBA 18% CaP	−0.7	123.8 ± 4.2	Yes
CBS 25 29% CaP	−0.7	48.9 ± 3.2	No
CBS 50 39% CaP	−0.8	79.4 ± 9.9	No
2:1 (P:L)			
CBS 25 40% CaP	−0.7	51.9 ± 8.3	Yes
CBS 50 49% CaP	−1.0	378.6 ± 23.1	Yes

**Figure 3:**
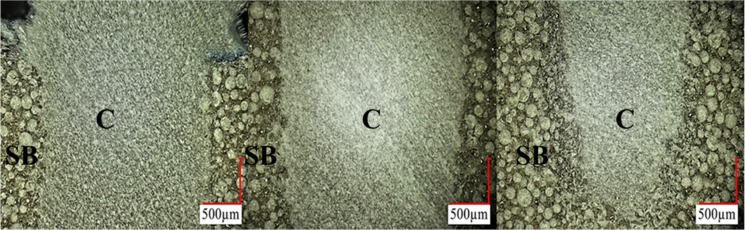
Flow and sealing effectiveness of the CBS 50 (40% filled) cement. The figure illustrates the material fully sealing a Sawbones^®^ defect of interest (2 mm × 10 mm) without leaving gaps along the interface using a rigid polyurethane foam model. The panels are arranged in order of increasing depth into the defect from left to right. No pressurization was necessary for the material to fill down toward the bottom of the defect. “SB” denotes Sawbones^®^, and “C” denoted cement.

Cement flow and the ability of the material to disperse and take the shape of a defect were characterized by injecting cement into a 2 mm × 10 mm pre-formed cylindrical defect. Figure 3 illustrates the sealing ability of the cement CBS 50 (40% filled). Microscopy revealed that the materials disperse well with no observable gaps or voids towards the bottom of the defect.

### 3.2. Rheology

**Figure 4:**
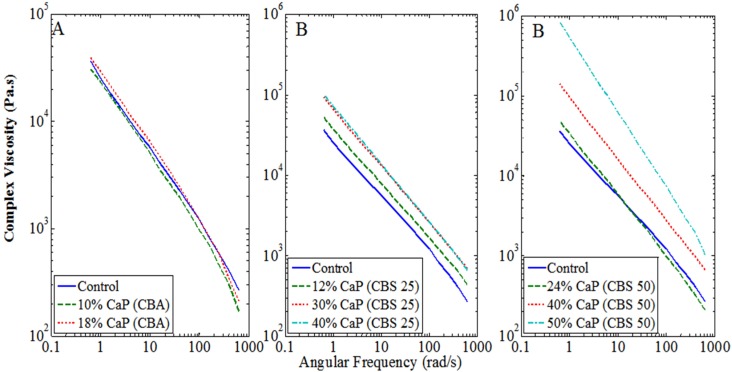
Complex viscosity of investigated materials over a range of angular frequencies (100–0.1 Hz). The shear-thinning effect of all cement compositions compared is demonstrated by the accentuated decrease in viscosity with increasing shear rate. (**A**) CBA materials *vs.* control showing no change in rheological characteristics among the formulations compared; (**B**) CBS 25 materials *vs.* control showing an increase in complex viscosity with an increase in filler concentration up to saturation. Note the curves for the 30% and 40% CBS 25 overlap; (**C**) CBS 50 materials *vs.* control showing a steady increase in complex viscosity with increasing concentration of filler in the material. It is interesting to note that although viscosity increased with these formulations, the materials’ pseudoplasticity also increased. Each of the formulations investigated other than the 50% filled CBS 50 resulted in significantly similar rheological characteristics in comparison to the control all-acrylic cement (*p* < 0.05).

**Figure 5:**
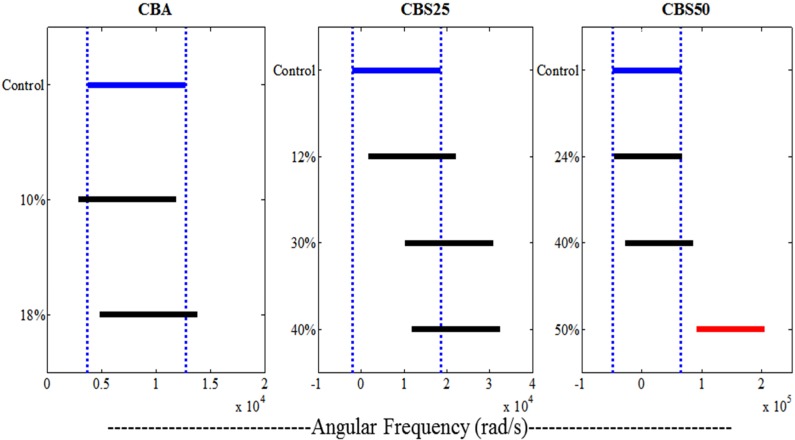
Analysis of Variance **(**ANOVA) values of the rheological data presented in Figure 4. Vertical dotted lines indicate the level of statistical significance. Samples which fall outside of these margins (CBS 50 panel) are statistically different across their means.

Figure 4A–C illustrates the results of the rheological analysis of CBA *versus* CBS materials in comparison to the standard all-acrylic formulation (control), with Figure 5 representing the statistical analysis of the results compared. The CBA materials are evaluated in Figure 4A, where the viscosity of the 10% and 18% (mass% filler, Table 1) formulations showed similar rheological behavior as compared to the control group (0% filler) (95% confidence, Figure 5). Figure 4B demonstrates that the CBS materials are much more efficient at incorporating the CaP into their formulation. Each of the CBS materials tested (12%–40% CaP filled CBS 25, Table 1) resulted in increased viscosity across the oscillatory frequencies tested, as compared to the 10%–18% CBA materials, illustrating the materials incorporation of the filler into its matrix. The curve representative of the 40% CaP filled CBS 25 material overlapped the 30% CaP filled CBS 25 curve highlighting the saturation of the filler at this concentration in the CBS cement (Figure 3B). Figure 4C outlines the CBS 50 material. The 24% filled material demonstrated negligible incorporation of filler (*p* > 0.05), while the 50% filled material demonstrates over-filling evidenced by the dramatic increase in negative slope. The 40% filled CBS 50 material retained the pseudoplastic characteristics of the control while still incorporating the filler into the matrix appropriately (Figure 4c).

### 3.3. Extrusion Stress (σ_extrusion_)

The extrusion stress of the cement formulations investigated ranged from ~23 Pa to ~400 MPa (Table 2). The control material (0% filler) demonstrated 116 ± 7.3 Pa, the CBA materials demonstrated 113 ± 15 Pa, the CBS 25 materials demonstrated 43 ± 14 Pa, and the CBS 50 materials demonstrated 161 ± 190 Pa.

### 3.4. Compressive Strength (Effect of CaP)

**Figure 6:**
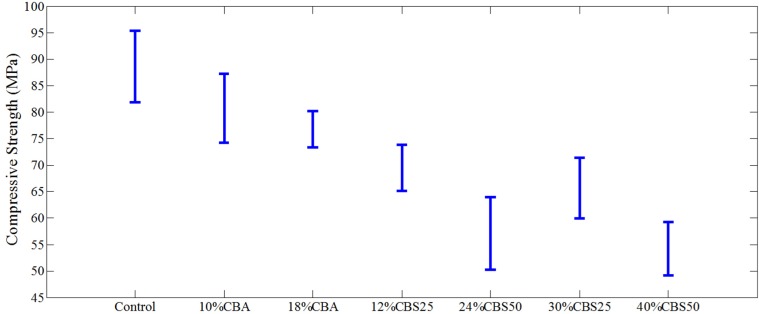
Compressive strength graphs of the materials investigated. The incorporation of CaP into the samples decreased the compressive strength of the materials investigated. The incorporation of CaP into the cement matrix resulted in more porous materials with decreased mechanical stability.

**Figure 7:**
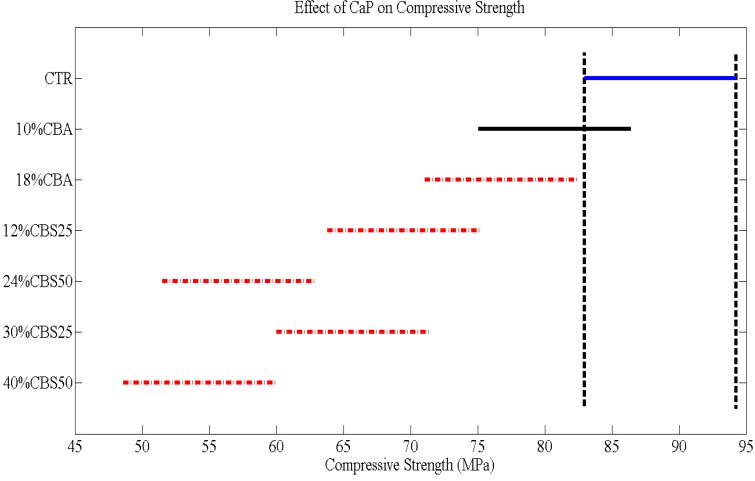
Analysis of Variance (ANOVA) values of the mechanical strength data presented in Figure 6. Vertical dotted lines indicate the level of statistical significance. Samples which fall outside of these margins are statistically different across their means.

Brushite was incorporated into the cement compositions at 0%–50% by mass. The compressive strength data illustrated in Figure 6 and Figure 7 demonstrates that the addition of Brushite additives at these concentrations did not significantly change the compressive strength of the CBA cement compositions up to 10% as compared to the control material (*p* > 0.05) (Figure 7). However, the 18% CBA material, as well as each of the CBS materials investigated, resulted in a steadily decreasing trend of compressive strength (Figure 6 and Figure 7). These materials were each significantly weaker (*p* < 0.05) than the control all-acrylic cement, although they were still within the range of implantable weight bearing materials.

## 4. Discussion

The overall goal of this study was to highly fill a two-solution cement system with calcium phosphate (Brushite) while maintaining the favorable pseudoplasticity and viscosity of the original material (control formulation, PMMA-based). The gel-like nature of two-solution PMMA-based cement enabled the variation of Brushite concentrations in the formulation in an effort to determine optimal filler concentrations within the system. It was noticed that the removal of PMMA beads resulted in decreased gel formation in the system. Therefore, this phenomenon was leveraged to allow for a higher concentration of filler to incorporate with the cement matrix without clumping or filter pressing, which are often associated with high calcium phosphate-filled cements.

The properties of bone cements are greatly affected by filler incorporation. Previous studies demonstrated that mixing calcium phosphates, radiopacifiers and antimicrobial compounds in powder-liquid cements can hinder the handling, mechanical strength and setting properties of these materials [12,13,14]. Particularly, calcium phosphates have been reported to degrade the handling of bone cements, making mixing and delivery of these materials challenging [8,9,10,11,12]. Thus, there are a number of factors which can influence the filling capacity of acrylic bone cements. The current study was interested in demonstrating the filling capacities of a pre-mixed two-solution system by varying the filler concentration with the acrylic polymer concentration. Results illustrated that to properly fill the acrylic cement system with Brushite, the acrylic phase (PMMA) must be partially substituted to allow adequate incorporation of the filler with the other components of the cement.

Cement viscosity is also influenced by a number of factors. There is an agreement within the literature that smaller particle size can result in increased viscosity of polymer systems because they tend to form particle networks that produce a yield phenomenon [27,28]. When adding CaP fillers in bone cements, several events can lead to an increase in viscosity and degradation of the material handling including: (1) increased particle volume in the mixture can lead to difficult wetting of the components of the cement mixture; (2) particle-particle interaction and network formation may lead to clumping of the filler phase; and (3) phase separation or filter-pressing [29]. To overcome some of the drawbacks provided by the addition of CaP fillers in powder-liquid cements, another study suggested a decrease in the P:L ratio and decrease in the plastic-limit of the powder besides decreasing particle size [6]. However, a decrease in the powder-to-liquid ratio is not recommended because it can induce significant changes in the cement thermal properties and residual monomer levels. It is important to note that no dispersant agents were necessary in the formulations investigated in this study. Additives may resolve injectability problems associated with powder-liquids cements containing high loads of CaP, however they can trigger changes in other properties such as mechanical integrity, thermal and setting behavior [6]. Brushite was incorporated in the two-solution cement system to overcome the lack of bioactivity observed with previously developed formulations [14,15,20]. The system presented in this study does not require any modification such as cross-linking or grafting of the polymer phase, but was simply based on optimization of the concentrations of the Brushite phase added to the mixture. The ultimate goal was to produce a unique bioactive, high-viscosity, pseudoplastic, and easily injectable PMMA-composite two-solution based bone cement for multiple uses in orthopedic and craniofacial surgery. This cement combines the mechanical strength of acrylic PMMA with the benefits of CaP compounds. Because CaP fillers are known to detrimentally degrade bone cement handling and injectability [4,5], it is critical to evaluate the effect of such fillers on the rheological characteristics when developing new formulations of bioactive cements. Brushite was selected for addition/substitution because it exhibits particular stability and resorption rates, which can be beneficial for different applications and patient needs. For example, a Brushite-loaded cement system would be partially resorbable due to the low Ca:P ratio of Brushite, allowing for a partially porous matrix for bone growth and interdigitation. Additionally, the CaP phase will be available for osteoblasts to utilize in an effort to create new bone at the defect site. Given the reported detrimental effects of such fillers in the handling and injectability characteristics of experimental and commercial cements [7,8,9,10,11,12], this study intended to demonstrate the ability of the two-solution system to incorporate the calcium phosphate phase. Clump formation was one parameter which gave insight into the de-mixing/filter pressing occurring with certain formulations (both CBAs and 50%-filled CBS 50) investigated (Table 2) [19]. This is a critical parameter to observe given that de-mixing of bone cements can impose complications to the patient due to the risk of delivering unreacted monomer *in vivo*. In the over-filled formulations investigated (those which exhibited clumping, Table 2), the last 1/4 to 1/3 of the cartridge required increased force to extrude the mixture. This observation is indicative of de-mixing or clumping. Each of the CBA materials investigated exhibited clumping (Table 2). Interestingly, these materials also showed no appreciable change in rheological behavior despite varying concentrations of filler. This is hypothesized to be a result of the CaP in the formulation being filter pressed out of the mixture during shearing. On the contrary, the CBS 25 materials showed no clumping or particle agglomeration in any composition prepared up to 30% CaP filler. The 40% filled CBS 25 material reached saturation, as illustrated in Figure 4B (same rheological behavior as the 30% filler), while also having clump formation (Table 2). The CBS 50 materials studied resulted in no clump prior to the powder-to-liquid ratio exceeding 1.5:1, as evidenced in Table 2.

These rheological characteristics are believed to be a result of the improved dispersion of the Brushite filler within the system [29]. The high shear-thinning (pseudoplastic) nature of these materials was illustrated by the negative slopes of the viscosity *versus* frequency curves (Figure 4A–C). The slopes of the regression lines (Figure 4A–C) give the type and degree of non-Newtonian flow, in which a zero slope would imply Newtonian behavior. The results revealed that the shear-thinning behavior was uncompromised by the addition of Brushite additives (CBA materials). The Brushite substituted materials (CBS materials) resulted in an increased shear-thinning behavior (when compared to control) as the concentration of Brushite was increased (Figure 4). This means the material is capable of being extruded from mixing nozzles and delivered to the location of interest, while upon removal of pressure, or at lower shear rates, the viscosity will recover exponentially. This is an important property because it will ensure ease of injection of highly viscous cements simply by adding moderate shear. High viscosity cements are desirable to stabilize cement flow, minimizing the risk of leakage [17]. However, the current challenge is that highly viscous cements will require forces for delivery that may approach or even exceed the human physical capability for injection [21]. The pseudoplasticity of the Brushite-filled cement system investigated is expected to facilitate flow and delivery through surgical needles or cannulas. Given the pseudoplasticity of these materials, viscosity is recovered once the cement is delivered to the treatment site, minimizing the risk for extravasation, which could potentially improve the outcomes of fracture treatment/implant augmentation via bone cement. Of the materials investigated, each of the CBS 50 materials (20%–50% filler) resulted in higher pseudoplasticity (when compared to control and CBA). These formulations demonstrated increased viscosity under low shear, and decreased viscosity under high shear as compared to the control and CBA formulations. Essentially, simply adding filler to the cement system was not efficient in incorporating the CaP phase because saturation prevented proper filler dispersion in the cement matrix. Therefore, it was observed that the substituted systems were more efficient in preventing saturation after filling (up to 40% CaP). Removing 25% of the dry polymer phase (CBS 25) allowed the system to fill up to 30% before saturation, while removing 50% of the dry polymer phase (CBS 50) allowed the system to fill up to 40% before dramatic changes to the material pseudoplasticity (Figure 4 and Figure 5).

The extrusion stress values of each composition were used to investigate the upper limit of filler concentration. This is a critical characteristic because it relates to the stress or pressure required to extrude or pump a material from its delivery system. Lower extrusion stress values are necessary to allow for manual extrusion of material through small gauge needle systems. The 40% filled CBS 50 material (80 Pa extrusion stress) has been demonstrated in this study to be easily injectable through a 20G needle with the use of manual pressure through the plunger. This is a highly desired characteristic for these cements. High pressure delivery devices can produce too low of a viscosity during injection, preventing the material from fully recovering its viscosity and facilitating extravasation. The control cement (prepared at a P:M ratio of 0.9:1) exhibited high viscosity, which is desirable for mitigation of extravasation. However, its high extrusion stress (115.5 ± 7.3) required the use of pressure driven devices to mix and inject the material. Besides, its low P:M ratio and high viscosity limited the addition of fillers or other compounds in the formulation. When Brushite was substituted for PMMA in the two-solution cement (CBS), the extrusion stress values dropped to a range of 26–80 Pa, while the added compositions (CBA) resulted in extrusion stress values of 102–124 Pa (relatively high for manual extrusion). Because of its prolonged swelling time within the delivery cartridge, the substituted two-solution composite cement reaches its doughy state prior to extrusion. Despite this, it is still within the normal range of stress values (the ability of the user to extrude the material manually) at its maximum viscosity (below 100 Pa). The CBS 50 material was the only material investigated which observed a significant increase in extrusion stress as the amount of filler increased. The 50% filled CBS 50 material resulted in an extrusion stress value of 378.6 ± 23.1 Pa. This was 254.8 Pa higher than the next highest extrusion stress recorded. This value was used to note the fill limit of the CBS 50 material.

The materials mechanical stability over the range of Brushite concentrations added was investigated to determine the effect of CaP incorporation on the cement compressive strength. It was demonstrated that increasing the Brushite concentration in the cement system decreased the compressive strength of the material. This is expected, due to the brittle nature of the CaP filler in comparison to the plastic PMMA phase. Results demonstrated that the CBS compositions were more effective in incorporating Brushite evidenced by the decreased compressive strength of the CBS 25 (12% CaP by mass) material compared to the 18% CaP-filled CBA material (Figure 6 and Figure 7). Compressive strength was investigated to ensure that after heavy CaP incorporation into the investigative cement materials, the mechanical integrity of the cements was still suitable for load-bearing applications. This property is of particular importance because in practice, these materials must provide mechanical strength to the locations they are administered to. The current study focused on the rheological characterization of Brushite-containing two-solution cements. It was demonstrated that optimal viscosities were achieved with 40% calcium phosphate filler. These cements exhibited adequate handling, injectability and strength. One of the experimental limitations of the current study was drying of the highly filled cements while loading and operating the rheometer. Also, this study was concerned specifically with the investigation of CaP filling effects on the cement rheology and compressive strength. Other cement parameters need to be fully investigated and characterized to provide information about optimal Brushite-containing two-solution cement compositions. Nevertheless, this study identified a series of CaP-filled cement formulations for additional characterization and development. A follow up study will report the ability of these Brushite-containing two-solution cements to incorporate and release antimicrobials. In that study the effect of filler concentration on the materials strength and setting have also been evaluated. Additionally, material cytocompatibility, exothermal behavior, residual monomer and *in vivo* integration are undergoing further evaluation. A study of the degradation rate of the Brushite phase will be conducted with *in vitro* experiments to determine the rate of pore generation within the cement composition. An ideal bone cement composition would maintain the original extrusion force throughout the cartridge rather than increase the force required during extrusion of the material while adequately incorporating the filler and maintaining material pseudoplasticity. Within this study, the most ideal composition identified in terms of viscosity, handling and extrusion stresses most closely resembles the CBS 50 (40% Brushite-filled). The composite two-solution materials investigated were demonstrated to be highly-filled, pseudoplastic, with viscosities that allow the material to fully fill sub-millimeter gaps/defects without pressurization.

## 5. Conclusions

In conclusion, this study investigated the CaP filling capabilities of a two-solution PMMA cement system. The highly-filled Brushite-containing cement system was designed to combine advantages of a CaP phase for improved interdigitation and osteoinduction along with the mechanical performance provided by an acrylic matrix. The two-solution cement system developed provides improved characteristics by allowing for complete dispersion and incorporation of fillers. This incorporation (up to 40% by mass Brushite) resulted in improved particle dispersion throughout the cement and prevented clumping or filter-pressing during cement extrusion. This resulted in viscous, highly injectable cements with pseudoplastic behavior and high compressive strength. Furthermore, such properties may qualify the cement as an effective alternative to conventional CaP-containing cements. The investigated cements are expected to find multiple applications in orthopedic and craniofacial surgery, providing improved interdigitation and integration with implants and bone.
